# Editor’s Introduction to ‘Personal perspectives in the physical sciences for the Royal Society’s 350th anniversary’

**DOI:** 10.1098/rsta.2009.0244

**Published:** 2010-03-13

**Authors:** M. Pepper

**Affiliations:** London Centre for Nanotechnology and Department of Electronic and Electrical Engineering, University College London, Torrington Place, London WC1E 6BT, UK

*Philosophical Transactions* has a long and distinguished history. As the oldest continuously published journal in the world, it is not surprising that many of the most famous names in physical science have published in it. To name just a few, Dirac, Faraday, Halley, Joule, Newton, Maxwell, Priestley, Rutherford and Young have all made contributions, which are now available on the web as with all the Royal Society publications. These days, the scope for a journal covering many subject areas is limited in view of the competition of specialized titles, consequently a few years ago the decision was made to eliminate contributed papers and devote each issue to a theme with a Guest Editor who would approach colleagues for articles. When combined with publishing the proceedings of the very popular discussion meetings held at the Royal Society, this has resulted in a very successful approach with the number of issues being increased from 12 to 24 per annum.

To commemorate the 350th anniversary of the Royal Society, this special issue has been published, which comprises a number of articles covering a spread of physical sciences in a manner not requiring the reader to be a specialist to gain understanding of the content. It is to be hoped that this will demonstrate the continued vitality and combination of relevance and longevity that characterize the journal.

